# 4-1BB immunotherapy: advances and hurdles

**DOI:** 10.1038/s12276-023-01136-4

**Published:** 2024-01-04

**Authors:** Rohit Singh, Young-Ho Kim, Sang-Jin Lee, Hyeon-Seok Eom, Beom K. Choi

**Affiliations:** 1 Immuno-oncology Branch, Division of Rare and Refractory Cancer, National Cancer Center, Goyang, 10408 Republic of Korea; 2 Diagnostics and Therapeutics Technology Branch, Division of Technology Convergence, Research Institute, National Cancer Center, Goyang, 10408 Republic of Korea; 3 Hematological Malignancy Center, National Cancer Center, Goyang, 10408 Republic of Korea; 4 Innobationbio, Co., Ltd., Mapo-gu, Seoul, 03929 Republic of Korea

**Keywords:** Tumour immunology, Immunotherapy

## Abstract

Since its initial description 35 years ago as an inducible molecule expressed in cytotoxic and helper T cells, 4-1BB has emerged as a crucial receptor in T-cell-mediated immune functions. Numerous studies have demonstrated the involvement of 4-1BB in infection and tumor immunity. However, the clinical development of 4-1BB agonist antibodies has been impeded by the occurrence of strong adverse events, notably hepatotoxicity, even though these antibodies have exhibited tremendous promise in in vivo tumor models. Efforts are currently underway to develop a new generation of agonist antibodies and recombinant proteins with modified effector functions that can harness the potent T-cell modulation properties of 4-1BB while mitigating adverse effects. In this review, we briefly examine the role of 4-1BB in T-cell biology, explore its clinical applications, and discuss future prospects in the field of 4-1BB agonist immunotherapy.

## Focus on 4-1BB immunotherapy

4-1BB is a costimulatory T-cell receptor expressed on activated T cells and natural killer cells. Targeting 4-1BB with agonistic antibodies is a promising immuno-oncology approach and has shown potential in preclinical animal models. However, the clinical use of agonistic 4-1BB antibodies for cancer immunotherapy has been hampered by adverse effects and insufficient anti-cancer activity. In this review, the authors provide an overview of the 4-1BB costimulatory pathways in T-cell-mediated cancer immunity and summarize ongoing efforts to develop improved therapies through 4-1BB stimulation. They also explore potential solutions to address the challenges encountered in clinical trials.

This summary was initially drafted using artificial intelligence, then revised and fact-checked by the author.

## 4-1BB and 4-1BB ligand

**Fig. 1:**
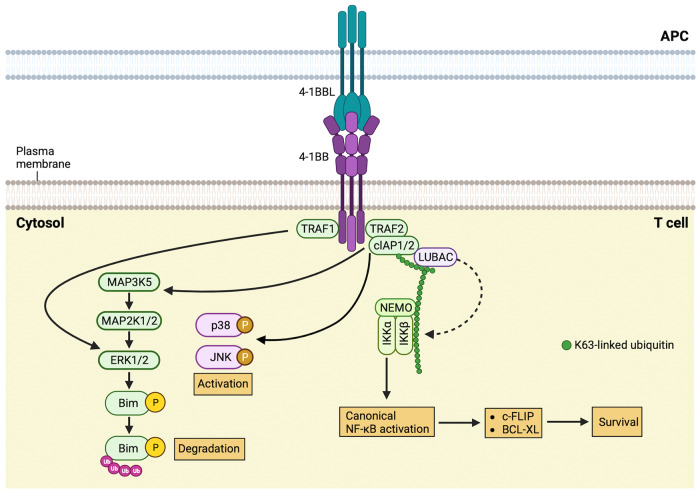
This illustration depicts the cascade of events involved in 4-1BB signaling and its effects on T-cell function. Upon interaction with 4-1BBL on antigen-presenting cells (APC), 4-1BB signaling begins by recruiting TRAF1 and TRAF2 to the TRAF-binding motif located in the cytoplasmic tail of 4-1BB, along with cIAP1 and cIAP2, forming the 4-1BB signaling complex. TRAF1 and TRAF2 lead to ERK1/2 activation through TRAF1 stimulation of ERK1/2 activity. The activation of ERK1/2 also induces the phosphorylation of the proapoptotic protein Bim. This phosphorylation event facilitates the proteasomal degradation of Bim, which, in turn, promotes T-cell survival. In the context of 4-1BB-induced NF-κB activation, the TRAF2-cIAP complex initiates the NF-κB pathway. The IKK complex comprises three subunits: two kinases, IKKα and IKKβ, along with the regulatory subunit NF-κB essential modulator (NEMO; also known as IKKγ). NF-κB induces the transcription of BCL-XL, BCL-2, and c-FLIP and stimulates the expression of IL-2, IL-4, and IFN-gamma, promoting the survival and cytotoxic activity of T cells. Additionally, TRAF2 recruitment fosters the activation of the JNK and p38 MAPK pathways. JNK translocates into the nucleus, where it interacts with the transcription factors JUN and ATF2, leading to the production of IL-2, IL-4, and IFN-gamma by T cells. TRAF TNF receptor-associated factor, cIAP cellular inhibitor of apoptosis protein, LUBAC linear ubiquitin chain assembly complex, NF-κB nuclear factor-κB, NEMO NF-κB essential modulator, IKK IκB kinase, JNK JUN N-terminal kinase.

4-1BB [also known as CD137 or TNF receptor superfamily member 9 (TNFRSF9)] was initially isolated in the 1980s using a differential screening method from mouse cDNA libraries and was later isolated from human peripheral T-cell cDNA libraries^1–3^. It was first described as an inducible molecule expressed on T cells after their activation^4,5^. 4-1BB is a glycosylated type I membrane protein and a member of the TNFR superfamily, consisting of four extracellular cysteine-rich pseudorepeat domains. The cytoplasmic region of 4-1BB contains a TNF receptor-associated factor (TRAF)-binding motif. In mice, 4-1BB is expressed in T cells, B cells, macrophages, natural killer (NK) cells, and dendritic cells (DCs); splenic DCs constitutively express 4-1BB^6^. Inducible expression of 4-1BB is primarily restricted to activated T cells. Similarly, in humans, 4-1BB expression is primarily found on the surface of activated cytotoxic CD8^+^ T cells and helper CD4^+^ T cells. Additionally, 4-1BB expression can be induced on NK cells, B cells, monocytes, and DCs upon activation. As an inducible costimulatory molecule, 4-1BB enhances the T-cell response to antigens. Upon upregulation and trimerization by 4-1BBL on T cells, 4-1BB recruits TRAF adaptor proteins to cytosolic TRAF-binding motifs, initiating costimulatory signaling^7,8^ (Fig. 1). TRAFs form homo or heterotrimers that serve as a scaffold, aiding in the assembly of the CD137 signalosome. Activation of 4-1BB signaling in T cells primarily enhances the survival of T cells by suppressing activation-induced cell death (AICD) through increased expression of antiapoptotic genes such as bcl-x(L), c-FLIP, bfl-1, and ERK-dependent Bim downmodulation^9–11^. Additionally, 4-1BB synergizes with CD28 costimulation to enhance cytokine production by activated T cells^12,13^. The cytosolic signaling domain of 4-1BB also contains a binding site for the T-cell-specific tyrosine kinase p56*lck*^14^. A recent comprehensive analysis of the 4-1BB signalosome by Glez-Vaz et al. using a combination of immunoprecipitation and mass spectrometry analysis has provided further insight into 4-1BB signaling^15^. They found that TRAF1, TRAF2, TRAF3, and TRAF5 physically interact with 4-1BB. More intriguingly, they discovered that cIAPs (cellular inhibitor of apoptosis proteins, specifically cIAP1 and cIAP2) are part of the 4-1BB signalosome. Although the role of cIAPs in 4-1BB signaling was suspected based on their role in other TNFR family signaling pathways, this study represents the first concrete evidence of their involvement in the 4-1BB signaling complex^15,16^.

The ligand for 4-1BB is 4-1BBL (also known as TNFSF9 or CD137L)^17,18^. 4-1BBL is a type II membrane protein of the TNF superfamily. In mice, 4-1BBL is constitutively expressed in splenic DCs and is upregulated on spleen germinal center B cells, macrophages, and antigen-presenting cells (APCs) after their activation^6,18^. The expression of 4-1BBL has also been detected in nonlymphoid epithelial cells, thymic fibroblasts, skin fibroblasts, and fibroblastic reticular cells of lymph nodes through RNA sequencing (The Immunological Genome Project)^19^.

In humans, 4-1BBL is expressed in T cells, B cells, DCs, and macrophages. Similar to the expression pattern in mice, 4-1BBL is also detected in nonlymphoid cells such as fibroblasts according to RNA sequencing (the Human Protein Atlas)^20^. 4-1BBL is also expressed on tumor cells of various origins, such as carcinoma and colon cancers^21,22^. Therefore, it is reasonable to speculate about the role of 4-1BB/4-1BBL signaling in shaping immunity in both lymphoid and nonlymphoid organs. In this review, we will focus on the role of 4-1BB/4-1BBL in lymphoid tissue and tumor immunity.

## Structure of the 4-1BB/4-1BBL complex

**Fig. 2:**
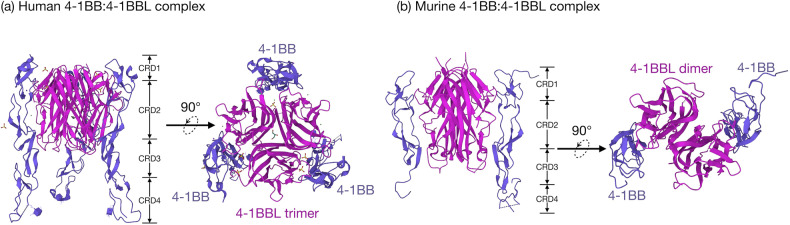
Representative structures of human and murine 4-1BB ligand‒receptor complexes. **a** Human 4-1BB/4-1BBL complex (PDB ID: 6CPR): This complex illustrates the structure of a 4-1BB receptor and its ligand in both side view (left) and top view (right)^24,27^. The 4-1BBL trimer is depicted in magenta, surrounded by three monomers of the 4-1BB receptor shown in blue. Notably, 4-1BBL binds to cysteine-rich domains (CRDs) 2 and 3 of the 4-1BB receptor. **b** Murine 4-1BB/4-1BBL complex (PDB ID: 6MKZ): This complex represents murine 4-1BBL in complex with its receptor 4-1BB, shown in side view (left) and top view (right)^25,28^. A dimeric 4-1BB ligand (magenta) binds externally to two 4-1BB monomers (blue). Each murine 4-1BBL binds to CRD1, 2, and 3 of the murine 4-1BB monomer, with CRD2 being the primary interaction site. The RCSB PDB 3D Protein Feature View was employed to create these molecular representations. PDB, Protein Data Bank.

The protein structure of TNFR family members is characterized by a single transmembrane domain, an extracellular domain with disulfide bond-stabilized cysteine-rich domains (CRDs) for ligand binding, and an intracellular signaling domain containing different motifs for signal transduction^23^. These CRDs form a characteristic ‘jellyroll’ fold consisting of two beta sheets held together by disulfide bonds (Fig. 2). Like some other TNFR family members, 4-1BB on human T cells exists in a disulfide-linked dimeric form^14,24^. In contrast, murine 4-1BB is primarily expressed in a monomeric form^25^. On the other hand, human 4-1BBL exists on the cell membrane as a homotrimer, whereas mouse 4-1BBL exists as a covalently linked dimer^26^. Human 4-1BB/4-1BBL binds in a heterohexameric conformation where the trimer formed by 4-1BBL binds with three molecules of 4-1BB^24,27^ (Fig. 2a). Each monomer in the 4-1BB dimer unit can bind separately to the 4-1BBL trimer. The receptor monomers bind to the outside at the interface between two 4-1BBL monomers. This allows the formation of a 2D lattice structure of 4-1BB and 4-1BBL molecules, facilitating strong 4-1BB signaling. Blocking the ability of 4-1BB to multimerize significantly suppresses 4-1BB signaling. In contrast, mouse 4-1BB/4-1BBL binds in a heterodimeric form where two 4-1BB receptor molecules bind with a 4-1BBL dimer^25,28^ (Fig. 2b). As 4-1BB signaling depends on the multimerization of receptor monomers to form an active signaling unit, this murine 4-1BB/4-1BBL confirmation does not appear to be sufficient to induce strong 4-1BB signaling. More recently, cooperation between 4-1BB, 4-1BBL, and galectins in the regulation of 4-1BB/4-1BBL signalosome formation has become evident. Galectin 9 is a lectin with conserved carbohydrate-recognition domains for β-galactosides. Galectin 9 supports the multimerization of 4-1BB monomers in the presence of a 4-1BB agonistic monoclonal antibody^29^. In Gal-9^−/−^ mice, the anti-4-1BB antibody loses its functionality of inducing T cells to upregulate IFN-γ production. Similarly, Gal-9^−/−^ T cells were less responsive to 4-1BBL-stimulated IFN-γ production^29^. A recent report described another galectin, galectin-3, that binds and modulates 4-1BB signaling^30^. In contrast to galectin 9, galectin 3 seems to be a negative regulator of 4-1BB signaling. Although galectin 3 does not hinder the binding of 4-1BB to either 4-1BBL or galectin 9, it promotes the shedding of membrane-bound 4-1BB, thus reducing 4-1BB signaling.

## 4-1BB is a metabolic regulator of T cells

Naïve T cells primarily rely on oxidative phosphorylation (OXPHOS) for their energy needs. Upon activation, either through encounters with foreign antigens such as viruses or tumor antigens, naïve T cells differentiate into memory T cells or effector T cells that proliferate and carry out antiviral or antitumoral functions by producing cytokines and other effector proteins. These metabolic changes are primarily regulated by T-cell receptor (TCR)-mediated phosphoinositide 3-kinase (PI3K) and Akt-mediated mTOR signaling^31^. Recent reports have implicated 4-1BB signaling in the regulation of T cell mitochondrial mass and functions^32–34^. 4-1BB costimulation in activated T cells triggers p38-MAPK signaling, which, in turn, activates ATF-2, mitofusin-2, and OPA-1, regulators of mitochondrial fusion and energetics^32–34^. This results in increased mitochondrial mass and respiratory capacity in T cells. On the other hand, we have demonstrated that ligation of CD137 using agonistic anti-4-1BB monoclonal antibodies activates glucose and fatty acid metabolism, supporting the increased energy and biomass demands for proliferation^35^. Anti-4-1BB antibody treatment in vitro upregulates the glucose transporter Glut1 on CD8^+^ T cells and activates the liver kinase B1 (LKB1)-AMP-activated protein kinase (AMPK)-acetyl-CoA carboxylase (ACC) signaling pathway. Notably, the AICD function of the anti-4-1BB antibody was compromised by the lipid metabolism inhibitor etomoxir but remained unaffected by the glycolysis inhibitor 2-deoxy-D-glucose (2-DG). Based on these data, it is tempting to speculate that during the immune activation phase, T cells might primarily use lipids for energy generation. This could have implications for the ex vivo amplification of T cells for immunotherapy.

## 4-1BB intracellular domain in CAR-T cells

Genetically engineered T cells expressing chimeric antigen receptors (CARs) have demonstrated remarkable therapeutic activity in patients with hematological malignancies^36^. CARs have a modular design consisting of four components: an extracellular antigen-binding domain, a hinge region, a transmembrane domain, and an intracellular signaling region containing costimulatory domains. The CD28 and 4-1BB-derived domains are the two most widely studied costimulatory domains for CAR T cells. Clinically approved CAR T cells currently use either of these two costimulatory domains^37,38^. Although CAR T cells containing either of these costimulatory domains exhibit comparable antitumor efficacy in patients with B-cell malignancies, preclinical models have shown substantial biological distinctions between CD28 and 4-1BB^39,40^. The activation of the 4-1BB signaling pathway promotes memory formation in CAR T cells, enhancing oxidative phosphorylation and mitochondrial biogenesis, which contributes to long-term immune memory against cancer cells^33^. The intracellular domain of CD28 also enhances CAR T-cell function by promoting T-cell activation, cytokine production, and proliferation. However, CD28 signaling primarily enhances the effector functions of CAR T cells, such as their immediate killing effect and short-term cytokine secretion, with enhanced glycolytic metabolism^41,42^. In contrast to CD28, the 4-1BB intracellular domain has been linked to a prolonged and enduring CAR T-cell response. This effect is achieved by mitigating exhaustion caused by persistent CAR signaling^41^. Sun et al.^43^ provided a possible explanation for this difference^43^. By comparing anti-CD19 CAR T cells with either the CD28 or 4-1BB costimulatory domain, they found that CAR T cells with the CD28 domain show antigen-independent CD3ζ phosphorylation and increased antigen-dependent T-cell activation even at low levels of antigen compared to CAR T cells with the 4-1BB costimulatory domain. This prolonged and chronic signaling in CD28 CAR T cells leads to exhaustion. CAR T cells incorporating the 4-1BB intracellular domain tend to exhibit improved persistence, long-term functionality, and memory formation. This characteristic of 4-1BB can be advantageous for achieving long-lasting antitumor effects and potentially reducing the likelihood of tumor relapse. Third-generation CAR T cells often incorporate both the 4-1BB and CD28 costimulatory domains to leverage the advantages offered by each domain: the cytotoxicity and proliferation of the CD28 domain with the enhanced memory and persistence of the 4-1BB domain^44^.

## 4-1BBL is a context-dependent regulator of T-cell activation or inhibition

4-1BBL is expressed in both lymphoid and nonlymphoid tissues. In lymphoid organs, 4-1BBL is an inducible ligand expressed on APCs such as DCs, B cells, and macrophages^18,45^. In nonlymphoid organs, 4-1BBL is expressed on keratinocytes, Schwann cells, and a wide range of tumor cells^21,22^. Although our understanding of the role of 4-1BBL in 4-1BB/4-1BBL signaling is still limited, there is increasing evidence suggesting that the stimulatory or inhibitory effect of the 4-1BBL ligand can vary depending on the cell type and context. Initial experiments with 4-1BBL-knockout mice revealed a role of 4-1BBL in allograft rejection and antiviral CD8^+^ T-cell responses^46^. In a lymphocytic choriomeningitis virus (LCMV) infection model, the absence of 4-1BBL resulted in a 2- to 3-fold decrease in the levels of CD8^+^ T cells compared to those in wild-type mice, although 4-1BBL-deficient mice still exhibited normal kinetics in generating cytotoxic T lymphocytes (CTL) and eliminating acute LCMV, suggesting that optimal CD8^+^ T-cell responses rely on interactions that depend on 4-1BBL^46^. Engagement of 4-1BBL has been shown to sustain T-cell survival and responses while enhancing cell division^47^. Recently, we demonstrated in a syngeneic tumor model that this observation holds true for self-tumor antigen-reactive CD8^+^ T cells as well^48^. Although 4-1BBL does not seem to play a critical role in early T-cell activation, it is indispensable for the maintenance of antitumor CD8^+^ T cells^48^.

Conversely, the engagement of 4-1BBL with its receptor, 4-1BB, has been demonstrated to induce inhibitory signals in T cells, resulting in the suppression of activation and proliferation. Croft and colleagues have shown that activated T cells upregulate both 4-1BB and 4-1BBL^49^. In tolerogenic conditions, T cells express elevated levels of 4-1BBL, along with 4-1BB. They propose that following T-cell activation in a tolerogenic environment, the upregulation of 4-1BB is not as potent as in an antigen-rich condition, while the upregulation of 4-1BBL is more pronounced. This enables 4-1BBL to remain on the surface of activated T cells and interact with 4-1BB-expressing APCs in a *trans* manner, ultimately leading to T-cell suppression. However, the mechanisms by which 4-1BBL transmits negative signals within T cells are still not fully understood.

## Hyperimmune environment in 4-1BB- and 4-1BBL-deficient mice: A puzzling question

The positive costimulation function of 4-1BB has been well established through various experiments using 4-1BB knockout and agonistic monoclonal antibodies. 4-1BB^−/−^ mice have shown reduced humoral responses and CTL activity in a vesicular stomatitis virus (VSV) infection model^50^. The anti-4-1BB agonist monoclonal antibody 3H3 induces rapid proliferation and reduces AICD in murine cells, both in vitro and in vivo. However, the same study documented enhanced T-cell functions in 4-1BB^−/−^ mice. T cells from 4-1BB-deficient mice exhibit hyperproliferative responses in vivo and in vitro in response to mitogens and anti-CD3 antibodies, respectively^50^. 4-1BBL^−/−^ mice have shown reduced CD8^+^ T-cell responses in influenza, LCMV, and gamma herpes virus models^51–55^. In humans, deficiencies in 4-1BB are mainly implicated in compromised antiviral CD8^+^ T-cell responses^56–58^.

In CD137^−/−^ MRL/MpJ-Tnfrs (lpr) mice, the onset of autoimmune lacrimal gland disease and systemic lupus erythematosus is significantly accelerated, and the severity of the disease increases as well; these changes are mediated by an increase in the levels of pathogenic CD4^+^ T cells^59,60^. In other studies, 4-1BB^−/−^ mice have shown enhanced clonal expansion of OT-I and OT-II CD8^+^ and CD4^+^ T cells, respectively^61,62^. Similarly, in a syngeneic mouse tumor model, 4-1BB^−/−^ mice exhibited an enhanced antitumor CD8^+^ T-cell response^63^. In a mouse cytomegalovirus (MCMV) infection model, 4-1BB^−/−^ mice showed elevated early CD8^+^ T-cell responses but reduced persistence of MCMV-specific memory CD8^+^ responses^64^. These findings suggest that the 4-1BB/4-1BBL pathway has dual regulatory functions, both stimulating and inhibiting signaling activities in vivo. However, the exact mechanisms underlying this duality are still not fully elucidated. One key challenge is the absence of cell-specific conditional knockout mice for 4-1BB and 4-1BBL. The availability of such mice would help minimize potential confounding effects or side effects associated with the global knockout of these genes. While our understanding of the role of 4-1BB/4-1BBL signaling in T cells has advanced, there is a crucial gap in our knowledge regarding the potential role of this signaling pathway in nonmyeloid tissues.

## 4-1BB/4-1BBL targeting for immunotherapy: *Cis*- or *trans*-interactions?

The conventional understanding of the 4-1BB/4-1BBL interaction is that 4-1BB is expressed on T cells and primarily interacts with 4-1BBL, which is expressed on APCs in a *trans*-interaction fashion^18^. However, to add complexity to the matter, there have been reports suggesting the expression of 4-1BBL on T cells as well^49,65^. Eun et al. have shown that both 4-1BB and 4-1BBL are expressed in T cells^49^. T cells from 4-1BB-knockout mice have greater expression levels of 4-1BBL than those from control mice after activation. This indicates that both 4-1BB and 4-1BBL are inducible proteins. However, 4-1BBL is hard to detect on normal and activated T cells. Thus, it is plausible that the levels of 4-1BB expression in T cells determine the 4-1BBL expression levels on the cell membrane via *cis*-interaction, followed by its internalization and subsequent degradation. Does this imply that there is a possibility of cis interaction between 4-1BB/4-1BBL? If so, what are the consequences of this interaction?

Recently, we attempted to answer these questions by using anti-4-1BB and anti-4-1BBL antibodies in combination with 4-1BB or 4-1BBL knockout CD8^+^ T cells^48^. We found that while the *trans*-interaction of 4-1BB and 4-1BBL has an impact on CD8^+^ T-cell survival, the *cis*-interaction of these molecules is crucial for supporting the survival of activated CD8^+^ T cells. Dawicki et al. previously found that the absence or deficiency of 4-1BBL in OVA-specific CD4^+^ (OT-II) and CD8^+^ T (OT-I) cells had little effect on their initial expansion after OVA immunization^65^. Our study similarly found that 4-1BBL deficiency in pmel-1 CD8^+^ T cells only slightly impacted their initial proliferation with self-antigen peptide mgp100 immunization^48^. However, when pmel-1 CD8^+^ T cells were stimulated with anti-4-1BB antibody, the difference in proliferation between wild-type and 4-1BBL-deficient pmel-1 CD8^+^ T cells was significant. Our findings suggest that the formation of the 4-1BB/4-1BBL complex in activated CD8^+^ T cells and the transmission of its signal are crucial, rather than the *trans*-interaction of 4-1BB and 4-1BBL between cells. Whether this holds true for human T cells is yet to be determined.

## 4-1BB/4-1BBL targeting for cancer immunotherapy and autoimmunity

Immunomodulation has proven to be an extremely successful strategy for treating a wide range of cancers. Broadly, immunomodulators can be classified into two categories: antagonists and agonists. The antagonist category includes antibodies that inhibit immunosuppressive molecules expressed either on immune cells or cancer cells to reinvigorate the antitumor immune function of the immune system, especially T cells. CTLA-4 (cytotoxic T-lymphocyte-associated protein 4 or CD152), PD-1 (programmed cell death 1), and PD-L1 (programmed death-ligand 1 or CD274) are well-established examples of targets of antagonist immunomodulators, and immune checkpoint inhibition therapy targeting these factors has been successful^66^. Additionally, several other molecules, such as TIM3 (T-cell immunoglobulin and mucin domain–containing protein 3), TIGIT (T-cell immunoreceptor with Ig and ITIM domains), LAG3 (lymphocyte activating 3), and others, are currently being investigated in clinical trials to determine their potential as targets for immune checkpoint inhibition therapy^67^.

The second class is agonistic immunotherapy, where certain proteins are expressed by activated immune cells, especially T cells, and agonistic antibodies against them can be used to induce the proliferation and survival of these activated immune cells, enhancing antitumor immunity. 4-1BB is an example of a receptor that can be targeted with an agonist immunomodulator^68^.

## Anti-4-1BB antibodies

**Fig. 3:**
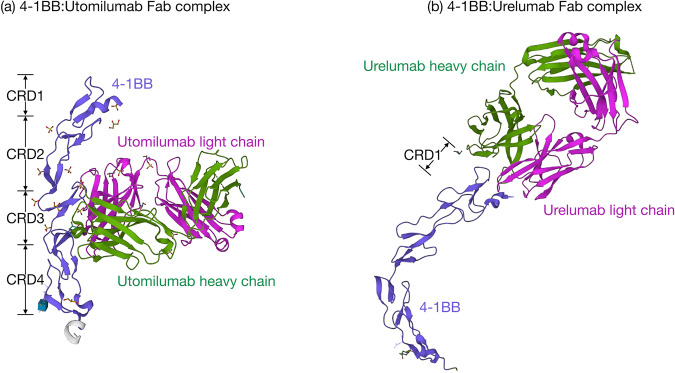
Structural comparison of 4-1BB binding by utomilumab and urelumab. **a** Utomilumab (heavy chain in green, light chain in magenta) binds alongside 4-1BB (blue), making contact at the junction between CRDs 3 and 4, thereby preventing the binding of 4-1BBL. This inhibition is likely due to steric occlusion (PDB ID: 6MI2)^75,76^. **b** Urelumab (heavy chain in green, light chain in magenta) binds to the very N-terminus of the 4-1BB (blue) receptor on CRD-1, which is separate from the 4-1BBL binding site located on CRD-2 and CRD-3. Urelumab does not interfere with subsequent 4-1BBL binding to the 4-1BB receptor (PDB ID: 6MHR)^75,77^. PDB, Protein Data Bank.

Agonistic antibodies for cancer therapy are an active field of research but have provided very few potential candidates for cancer immunotherapy^69^. Targeting 4-1BB using agonistic antibodies is currently a main focus of research for immune cell activation^70^. Although it is now clear that anti-4-1BB antibodies are excellent therapeutic agents in inhibiting tumor growth in various in vivo mouse models, a major limitation is that a strategy is still needed to safely translate anti-4-1BB immunotherapy into the clinic with a manageable toxicity profile^71,72^. After initial setbacks, over the past few years, there has been a reignited interest in 4-1BB targeting for cancer immunotherapy. To date, two agonistic anti-human 4-1BB antibodies have entered phase I/II clinical trials. The first one was the fully humanized IgG4 antibody, urelumab (BMS-663513, ClinicalTrials.gov Identifier: NCT02534506), and the second one was a humanized IgG2 antibody, utomilumab (PF-05082566, ClinicalTrials.gov Identifier: NCT01307267). A clinical trial conducted with the anti-4-1BB mAb urelumab was hampered by a high incidence of grade 4 hepatitis^73^. In another clinical trial conducted with utomilumab, the antibody was well tolerated but had relatively low efficacy^74^. Utomilumab binds to the CRD3 and CRD4 domains of 4-1BB and overlaps with the 4-1BBL binding site, thus abrogating the subsequent binding of 4-1BBL to 4-1BB^75,76^ (Fig. 3a). Urelumab binds to the end of the N-terminus of the 4-1BB receptor on CRD-1, away from the 4-1BBL binding site on 4-1BB, it thus does not block the 4-1BB and 4-1BBL interaction^75,77^ (Fig. 3b). Therefore, it is possible that these antibodies exhibit different properties due to their functional differences. The effective signaling of human TNFRSF superfamily receptors typically requires the formation of complexes with their ligands, followed by hyperclustering. In the case of utomilumab, as it inhibits the 4-1BB/4-1BBL interaction, the antibody itself may not be sufficient to induce the hyperclustering of 4-1BB molecules, rendering it functionally inert^78^.

Extensive research is currently focused on modulating the functions of 4-1BB antibodies^79,80^. A recent study by Yu et al. presents a strategy in this regard^80^. The authors leveraged the understanding that high-affinity antibodies can impede receptor oligomerization and activation, potentially limiting their agonistic potential compared with low-affinity versions of the same antibody^81^. Building upon this hypothesis, they generated a lower affinity variant of utomilumab, transforming it from an inert antibody into a potent agonistic antibody^80^. Remarkably, this lower affinity version also demonstrated reduced antibody-dependent cellular phagocytosis (ADCP) and antibody-dependent cellular cytotoxicity (ADCC), which are undesirable properties for an agonistic antibody. If these findings hold true, they could pave the way for the development of a safer and more effective anti-4-1BB antibody. It will be intriguing to observe how this antibody performs in clinical trials.

In addition to urelumab and utomilumab, a new generation of 4-1BB agonistic antibodies with distinct mechanisms of action is in various phases of development. EU101 (ClinicalTrials.gov Identifier: NCT04903873), a humanized IgG1 antibody, is in phase I/II clinical trials. Both AGEN2373 (ClinicalTrials.gov Identifier: NCT04121676) and ADG206 (ClinicalTrials.gov Identifier: NCT05614258) are human IgG1 antibodies but differ in their affinity for FcγRs^82,83^. AGEN2373 binds to CRD4 and thus does not interfere with 4-1BBL binding^82^. HOT-1030 (ClinicalTrials.gov Identifier: NCT05060263), a human IgG1 antibody, binds to CRD1 and can cluster 4-1BB receptors in a superagonistic manner, independent of FcγR binding, potentially avoiding liver toxicity induced by FcγRIIB crosslinking^70^.

Another group comprises human IgG4 antibodies that rely on functional Fcγ-receptor cross-linking. Examples of such antibodies include ADG106 (NCT3802955), ATOR-1017 (NCT04144842), PE0116 (CTR20201794), and CTX-471 (NCT03881488), which differ from urelumab and utomilumab in their epitope recognition and 4-1BBL-blocking properties^83–86^. ADG106, ATOR-1017, and PE0116 antibodies block the 4-1BB/4-1BBL interaction, whereas CTX471 binds to the CRD3 and CRD4 domains of 4-1BB and does not block its interaction with 4-1BBL. Another approach relies on IgG4 antibodies such as LVGN6051 (NCT04130542) and STA551 (EudraCT: 2019–003329-11, JapicCTI-205153), which have Fc mutations to enhance Fcγ-receptor RIIB binding and reduce competition with endogenous IgGs^79,87^. Additionally, these antibodies possess weaker agonistic binding or bind to 4-1BB at high ATP concentrations only available in the tumor microenvironment to minimize systemic effects^79,87^.

## Engineered proteins

The success of aflibercept and blinatumomab opened up a whole new field of engineered protein-based therapeutics^88,89^. To overcome systemic toxicity and improve the efficacy of 4-1BB immunotherapy, some groups have explored this protein engineering approach to harness 4-1BB costimulation by designing multivalent proteins. The basic principle behind these designs is to retain the 4-1BB-stimulating arm while adding selectivity toward tumors to mitigate systemic cytotoxicity induced by strong 4-1BB stimulation. To minimize Fcγ-receptor cross-linking-induced hepatotoxicity, these recombinant proteins often use a modified Fc region. Bispecific antibodies, namely, MCLA-145 and GEN1046, have been developed to target both 4-1BB and PD-L1 simultaneously, while GEN1042 targets 4-1BB and CD40 concurrently^90–92^. In terms of hepatotoxicity, these antibodies have demonstrated improved outcomes compared to first-generation anti-4-1BB antibodies, as observed in their preclinical characterization studies and phase I trials^70^.

You et al. developed a recombinant bispecific antibody-like protein in which one arm targets B7-H3 and the other arm targets 4-1BB^93^. This design allows for enhanced localization of the protein at the tumor site, thereby reducing the side effects associated with systemic distribution of the anti-4-1BB arm. Encouraging in vivo results have been obtained, warranting further evaluation in human clinical trials to determine the effectiveness of this approach. In contrast, Claus et al. pursued a distinct strategy by creating a bispecific protein consisting of a trimeric 4-1BBL portion that engages 4-1BB on T cells, linked to a binding site for fibroblast activation protein α (FAP) or anti-CD19^94^. Additionally, the Fc region of the protein was mutated to prevent FcγR-mediated cross-linking. The FAP-binding site facilitates the localization of the protein to FAP-expressing tumor sites, while the trimeric 4-1BBL provides costimulation to T cells. Melero et al. recently conducted a first-in-human clinical study utilizing this recombinant protein and demonstrated the feasibility of this approach^95^. The available data show promising results, and it will be interesting to observe the performance of this approach in advanced clinical trials.

## Concluding remarks and future directions

4-1BB is a highly potent costimulatory molecule expressed in T and NK cells. Due to their unique ability to stimulate cytotoxic T-cell responses, 4-1BB agonists are receiving substantial attention as promising strategies for improving cancer immunotherapy^70,96^. One major question related to immunotherapy using anti-4-1BB agonistic antibodies is whether their primary site of action is the tumor tissue itself, where they amplify tumor antigen-reactive T cells, or if their effects are mainly observed in the tumor-draining lymph nodes, where they enhance the activation of newly encountered T cells in the presence of tumor antigen-loaded dendritic cells. Our in vivo data suggest that the latter scenario is more likely^97^. We observed compromised antitumor functionality of the anti-4-1BB antibody when we blocked T-cell egress from the lymph nodes using the S1PR1-alpha blocker FTY720, which hinders lymphocyte egress from lymphoid organs^97,98^. Supporting this hypothesis, two other well-executed studies have shown that the tumor-draining lymph nodes serve as the major site of action for immunomodulatory antibodies^99,100^. Considering these findings, when designing recombinant proteins targeting the 4-1BB axis, we should consider whether the protein will solely localize to the tumor tissue or if it will also be available to amplify T-cell responses in the tumor-draining lymph nodes, as demonstrated by the FAP–4-1BBL developed by Claus et al^94^.

The optimal combination of immune-modulating mAbs represents a promising avenue for advancing cancer immunotherapy. A compelling strategy involves pairing mAbs that play distinct roles in the immune response. For instance, one effective approach could involve combining a mAb that amplifies the activity of preexisting tumor-reactive T cells, such as a 4-1BB agonist or a PD-1/PD-L1-blocking antibody, with another mAb that diversifies the pool of tumor-reactive T cells, such as a CTLA-4 blocking-antibody. However, given that CTLA-4 blockade has exhibited limited efficacy, there is a need to develop innovative mAbs capable of expanding the diversity of tumor-reactive T cells. Furthermore, the simultaneous use of a mAb that enhances T-cell responses and one that augments the antigen-presenting function of APCs holds considerable promise^92,100–102^. This dual strategy aims not only to increase the number of tumor-reactive T cells but also to optimize their activation and function by enhancing antigen presentation. The inclusion of mAbs targeting CD47, SIRPa, and VSIG-4, which can modulate APC function, further enriches this strategy^103–107^.

Various bispecific mAbs, many of which target 4-1BB and other immune-related molecules, have recently emerged in the field of cancer immunotherapy^70^. While these bispecific mAbs offer a novel avenue for immune modulation, questions remain regarding whether their dual-targeting capabilities will enable them to outperform the combination of two distinct mAbs in terms of efficacy. Active clinical trials will be required to assess the comparative benefits and potential limitations of both approaches. These investigations are poised to provide valuable insights into the future landscape of cancer immunotherapy and the most effective strategies for harnessing the immune system’s potential against cancer.
